# DNA Evidence for Strong Genome-Wide Pleiotropy of Cognitive and Learning Abilities

**DOI:** 10.1007/s10519-013-9594-x

**Published:** 2013-04-23

**Authors:** Maciej Trzaskowski, Oliver S. P. Davis, John C. DeFries, Jian Yang, Peter M. Visscher, Robert Plomin

**Affiliations:** 1 MRC Social, Genetic & Developmental Psychiatry Centre, Institute of Psychiatry, King’s College London, DeCrespigny Park, Denmark Hill, London, SE5 8AF UK; 2 University of Colorado, Institute for Behavioral Genetics, Boulder, CO, 80309 USA; 3 University of Queensland Diamantina Institute, Princess Alexandra Hospital, The University of Queensland, Brisbane, QLD 4102 Australia; 4 Queensland Brain Institute, The University of Queensland, Brisbane, QLD 4072 Australia

**Keywords:** Pleiotropy, Intelligence, Learning abilities, Mathematics, Language, GCTA, Twins, Heritability, Cognition

## Abstract

Very different neurocognitive processes appear to be involved in cognitive abilities such as verbal and non-verbal ability as compared to learning abilities taught in schools such as reading and mathematics. However, twin studies that compare similarity for monozygotic and dizygotic twins suggest that the same genes are largely responsible for genetic influence on these diverse aspects of cognitive function. It is now possible to test this evidence for strong pleiotropy using DNA alone from samples of unrelated individuals. Here we used this new method with 1.7 million DNA markers for a sample of 2,500 unrelated children at age 12 to investigate for the first time the extent of pleiotropy between general cognitive ability (aka intelligence) and learning abilities (reading, mathematics and language skills). We also compared these DNA results to results from twin analyses using the same sample and measures. The DNA-based method revealed strong genome-wide pleiotropy: Genetic correlations were greater than 0.70 between general cognitive ability and language, reading, and mathematics, results that were highly similar to twin study estimates of genetic correlations. These results indicate that genes related to diverse neurocognitive processes have general rather than specific effects.

## Introduction

Very different neurocognitive processes appear to be involved in cognitive abilities such as reasoning and mathematics (Deary 2000) However, quantitative genetic research, largely based on twin studies, consistently indicates that genes that affect individual differences in performance in one domain are largely the same genes that affect performance in other domains, leading to the Generalist Genes Hypothesis (Plomin and Kovas 2005).

It is now possible to use DNA itself to estimate genetic influence in any sample of unrelated individuals rather than relying on comparisons between monozygotic and dizygotic twins. The method, implemented in a tool called Genome-wide Complex Trait Analysis (GCTA; Yang et al. 2011a) does not attempt to identify specific genes associated with traits. Instead, it correlates genomic similarity across hundreds of thousands of single nucleotide polymorphisms (SNPs) with phenotypic similarity in a large sample of unrelated individuals (Yang et al. 2010). This population-based approach does not rely on the strong assumptions made in classical twin studies.

Univariate Linear Mixed Model (LMM) implemented in the GCTA package has been used to estimate genetic influence for height and body mass index (Yang et al. 2010, 2011b), psychiatric and medical disorders (Lee et al. 2011), personality (Vinkhuyzen et al. 2012), and cognitive abilities (Davies et al. 2011; Plomin et al. 2013b). In contrast to univariate genetic analysis, bivariate genetic analysis focuses on the genetic correlation, the correlation between genetic influences on different traits, called pleiotropy (Plomin et al. 2013a). High genetic correlations between phenotypes are often interpreted as an indication that the same genes affect the phenotypes. Genetic correlations between diverse cognitive abilities as estimated through twin studies are typically greater than 0.60, indicating that cognition-related genes largely have general pleiotropic effects (Calvin et al. 2012; Plomin and Kovas 2005). However, the genetic correlation estimated from twin studies could be biased due to misspecification of the model of twin similarity for genetic and non-genetic effects. In this study, we use the GCTA package to estimate the genetic correlation between traits in conventionally unrelated individuals based on DNA evidence alone; this estimate is free of bias if we assume that the sole reason for phenotypic similarity between conventionally unrelated individuals is shared additive genetic factors. For brevity, we refer to LMM used in the GCTA package simply as GCTA.

Here we use bivariate GCTA (Lee et al. 2012; Yang et al. 2011a) to test the Generalist Genes Hypothesis by estimating genetic correlations between general cognitive ability (‘g’, aka intelligence) and language, reading, and mathematics. We compare these genetic correlation estimates from GCTA to those obtained from the twin design using the same sample assessed at the same age with the same measures. We also analyze the variables of height and weight for purposes of comparison.

## Materials and methods

### Sample and genotyping

The sample was drawn from the Twins Early Development Study (TEDS), which is a multivariate longitudinal study that recruited over 11,000 twin pairs born in England and Wales in 1994, 1995 and 1996 (Haworth et al. 2012; Oliver and Plomin 2007). TEDS has been shown to be representative of the UK population (Kovas et al. 2007). The project received approval from the Institute of Psychiatry ethics committee (05/Q0706/228) and parental consent was obtained prior to data collection.

Cognitive and DNA data were available for 3,747 11- and 12-year-old children whose first language was English and had no major medical or psychiatric problems. From that sample, 3,665 DNA samples were successfully hybridized to Affymetrix GeneChip 6.0 SNP genotyping arrays using standard experimental protocols as part of the WTCCC2 project (for details see Trzaskowski et al. 2013). In addition to nearly 700,000 genotyped SNPs, more than one million other SNPs were imputed from HapMap 2, 3 and WTCCC controls using IMPUTE v.2 software (Howie et al. 2009). 3,152 DNA samples (1,446 males and 1,706 females) survived quality control criteria for ancestry, heterozygosity, relatedness, and hybridization intensity outliers. To control for ancestral stratification, we performed principal component analyses on a subset of 100,000 quality-controlled SNPs after removing SNPs in linkage disequilibrium (r^2^ > 0.2) (Fellay et al. 2007). Using the Tracy–Widom test (Patterson et al. 2006), we identified 8 axes with *p* < 0.05, which were used as covariates in GCTA analyses.

The mean age of the sample was 11.5 years (SD = 0.66). The sample sizes for the GCTA results shown in Table 1 are 2,325 for ‘g’ and language, 2,238 for ‘g’ and mathematics, 2,250 for ‘g’ and reading, and 2,296 for height and weight. For the twin analyses, cognitive data were available for 5,434 twin pairs (Davis et al. 2009); however, the twin analyses presented here were based only on twins included in the GCTA analyses in order to provide a more precise comparison between GCTA and twin-study results. The numbers of twin pairs were 2,205, 2,095, 2,104 and 2,162, respectively.

**Table 1:** Genome-wide Complex Trait Analysis (GCTA) and twin study estimates of genetic correlations. Standard errors (SE) are shown in parentheses. ‘g’ refers to general cognitive ability

	Genetic correlation	
Bivariate comparison	GCTA (SE)	Twin (SE)
‘g’ vs language	0.81 (0.15)	0.80 (0.06)
‘g’ vs mathematics	0.74 (0.15)	0.73 (0.03)
‘g’ vs reading	0.89 (0.26)	0.66 (0.05)
‘g’ vs height	−0.13 (0.30)	−0.03 (0.06)
‘g’ vs weight	−0.04 (0.25)	−0.06 (0.06)
Height vs weight	0.76 (0.13)	0.65 (0.02)

### Measures

Cognitive data were collected online via the Internet using, where possible, adaptive branching, which enabled measurement of the full range of ability using a relatively small number of items. Details about the following measures, including references, are available elsewhere (Kovas et al. 2007).

#### General cognitive ability (g)

‘g’ was assessed from two verbal tests and two non-verbal tests. The verbal tests included WISC-III-PI Multiple Choice Information (General Knowledge) and Vocabulary Multiple Choice subtest. The two non-verbal reasoning tests were WISC-III-UK Picture Completion and Raven’s Standard and Advanced Progressive Matrices.

#### Language

Three components of language were assessed: syntax, semantics and pragmatics. Syntax was measured using the Listening Grammar subtest of the Test of Adolescent and Adult Language. Semantics was assessed using Level 2 of the Figurative Language subtest of the Test of Language Competence. Pragmatics was assessed using Level 2 of the Making Inferences subtest of the Test of Language Competence.

#### Mathematics

Assessment of mathematics targeted three components of mathematics: Understanding Number, Non-numerical Processes, and Computation and Knowledge. The items for these three scales were based on the National Foundation of Educational Research 5–14 Mathematics Series.

#### Reading

Four measures of reading were employed. Two measures assessed reading comprehension: the reading comprehension subtest of the Peabody Individual Achievement Test and the GOAL Formative Assessment in Literacy for Key Stage 3. Reading fluency was assessed by an adaptation of the Woodcock–Johnson III Reading Fluency Test and by the Test of Word Reading Efficiency, which was administered by telephone.

Composite measures for ‘g’, language, mathematics, and reading. For each cognitive measure, outliers above or below 3 SD from the mean were excluded. Scores were regressed on sex and age, and standardized residuals were derived and quantile normalized (Lehmann 1975; van der Waerden 1975). Composite measures for ‘g’, language, mathematics, and reading were created as unit-weighted means requiring complete data for at least 3 of the 4 tests for ‘g’ and reading and 2 of 3 tests for language and mathematics. All procedures were executed using R *(* www.r-project.org; R Development Core Team 2011 *)*. The phenotypic correlations among the composite measures were 0.63 for ‘g’ and language, 0.63 for ‘g’ and mathematics, and 0.57 for ‘g’ and reading.

#### Height and weight

Height and weight were assessed on the same sample (age 12) via self-report. Similar to the cognitive measures, outliers (± 3SD) were removed and scores were controlled for age and sex. The phenotypic correlation between height and weight was 0.63.

### Statistical analyses

#### GCTA

Conceptually, the amount of phenotypic variance, or covariance, explained by genetic factors is estimated by a comparison of a matrix of pairwise genomic similarity to a matrix of pairwise phenotypic similarity (Yang et al. 2010). Before the variance or covariance can be decomposed into genetic and residual components, we need to calculate pairwise genomic similarity between all pairs of individuals in the sample using all genetic markers genotyped on the SNP array. Because the GCTA package uses a random effects model to estimate genetic effects from a sample of unrelated individuals in the population, any pair whose genetic similarity is equal to or greater than a fourth cousin is removed (estimate of pairwise relatedness >0.025). In univariate analysis, the variance of a trait can be partitioned using residual maximum likelihood into genetic and residual components. Detailed description of this method can be found in GCTA publications (Yang et al. 2010, 2011a, b). The bivariate method extends the univariate model by relating the pairwise genetic similarity matrix to a phenotypic covariance matrix between traits 1 and 2 (Lee et al. 2012). The eight principal components described earlier were used as covariates in our bivariate GCTA analyses; as mentioned in the previous section, all phenotypes were age- and sex-regressed prior to analysis.

Twin modelling. The twin design and model-fitting is discussed elsewhere (Plomin et al. 2013a). We fit a bivariate Cholesky decomposition using OpenMx (Boker et al. 2011), which provided a direct comparison with the bivariate GCTA. The correlated factor solution is the least restricted model allowing variables to correlate with one another via genetic, shared environment, and non-shared environment. Because previous analyses of these data indicated nonsignificant differences in model-fitting results between males and females (Kovas et al. 2007), we combined same-sex and opposite-sex DZ twin pairs in order to increase the power of the analyses.

## Results

Table 1 shows GCTA-estimated genetic correlations (and standard errors, SE) between ‘g’ and learning abilities for more than 2,238 12-year-old UK twins (randomly selecting only one member of each twin pair to control for potential confounds, such as birth order) based on 1.7 million SNPs measured from the Affymetrix 6.0 GeneChip or imputed from HapMap 2,3 and WTCCC controls (Trzaskowski et al. 2013). Genetic correlations are significant and substantial for all three comparisons—between ‘g’ and language (0.81), mathematics (0.74), and reading (0.89). The GCTA-estimated genetic correlations between ‘g’ and learning abilities are similar in magnitude to the GCTA-estimated genetic correlation between height and weight (0.76). In addition, Table 1 includes bivariate results for ‘g’ versus height and ‘g’ versus weight as ‘negative controls’; their phenotypic correlations are both 0.07. As expected, these comparisons yielded negligible and nonsignificant genetic correlations (−0.03 and −0.06, respectively).

Table 1 also includes analogous genetic correlations from twin model-fitting analyses, as estimated from the same twin sample but including the co-twins (more than 2,095 pairs of twins). The GCTA-estimated genetic correlations are highly similar to the twin study estimates and do not differ significantly, as indicated by their overlapping standard errors. The similarity of GCTA and twin estimates of genetic correlations extend to the comparison between height and weight as well as the negative control comparisons of ‘g’ and height and ‘g’ and weight.

Tables 2 and 3 show full results from the bivariate GCTA and twin analyses, respectively.

**Table 2:** Bivariate Genome-wide Complex Trait Analysis (GCTA) results (with standard errors) for general cognitive ability (‘g’) versus language, mathematics, and reading, as well as comparison data for: g and height, g and weight, and height and weight

Variables	A						E				Vp_tr1	Vp_tr2	n
	V(G)_tr1	V(G)_tr2	C(G)_tr12	V(G)/Vp_tr1	V(G)/Vp_tr2	r_G_	V(e)_tr1	V(e)_tr2	C(e)_tr12	r_E_^a^			
‘g’ vs language	0.36(0.14)	0.35(0.14)	0.29(0.12)	0.37(0.14)	0.35(0.14)	0.81(0.15)	0.63(0.14)	0.65(0.14)	0.33(0.12)	0.52(0.11)	0.99(0.03)	1(0.03)	2325
‘g’ vs maths	0.36(0.13)	0.32(0.12)	0.25(0.10)	0.36(0.13)	0.32(0.12)	0.74(0.15)	0.64(0.13)	0.67(0.12)	0.38(0.10)	0.57(0.09)	0.99(0.03)	1(0.03)	2238
‘g’ vs reading	0.34(0.13)	0.16(0.12)	0.20(0.10)	0.34(0.13)	0.16(0.12)	0.89(0.26)	0.65(0.13)	0.84(0.12)	0.36(0.10)	0.49(0.09)	0.99(0.03)	1(0.03)	2259
‘g’ vs height	0.34(0.14)	0.36(0.15)	−0.05(0.10)	0.35(0.14)	0.36(0.14)	−0.13(0.30)	0.65(0.14)	0.64(0.14)	0.11(0.10)	0.17(0.16)	0.99(0.03)	1(0.03)	1868
‘g’ vs weight	0.35(0.14)	0.47(0.15)	−0.02(0.10)	0.35(0.14)	0.47(0.14)	−0.04(0.25)	0.64(0.14)	0.53(0.14)	0.04(0.10)	0.07(0.17)	0.99(0.03)	1(0.03)	1868
height vs weight	0.37(0.15)	0.49(0.15)	0.33(0.12)	0.37(0.14)	0.48(14)	0.76(0.13)	0.63(0.14)	0.52(0.14)	0.32(0.12)	0.56(0.12)	1.0(0.03)	1(0.03)	2286

GCTA incorporates full-information maximum likelihood that uses the full sample of more than 2,900 individuals with data on trait 1 or trait 2. However, the variance estimates for each trait are based on individuals with data for that trait and the covariance estimates are based on individuals with data for both traits. The n reported in the last column is the most conservative, i.e., the n that was used for the estimation of the covariance *V(G)* variance explained by genetic factors for trait 1 and trait 2 (tr1, tr2), *C(G)* covariance between trait 1 and 2 explained by genetic factors; *V(e)* residual variance for trait 1 and trait 2, *C(e)* residual covariance between trait 1 and trait 2; *Vp* phenotypic variance for trait 1 and trait 2, *V(G)/Vp* proportion of the phenotypic variance explained by genetic factors for trait 1 and trait 2, *r*_*G*_ genetic correlation between trait 1 and trait 2; *logL* log likelihood estimation of the model, *n* number of individuals with data for both trait 1 and trait 2, values in parentheses are standard errors

^a^The current version of GCTA does not report the environmental correlation or its standard error. The environmental correlation was derived here from the GCTA estimates using the following algorithm: C(e)_tr12/(√V(e)_tr1 × √V(e)_tr2), whereas the standard error was calculated using: Var(re) = re  ×  re  ×  (VarVe1/(4 × Ve1 × Ve1) + VarVe2/(4 × Ve2 × EV) + VarCe/(Ce × Ce) + CovVe1Ve2/(2 × Ve1 × Ve2 − CovVe1Ce/(Ve1 × Ce) − CovVe2Ce/(Ve2 × Ce)); SE(re) = sqrt[Var(re)], where re is the environmental correlation, Ve1 is the residual variance for trait 1, Ce is the residual covariance between two traits, VarVe1 is the sampling variance for Ve1 (residual variance for trait 1), VarCe is the sampling variance for Ce, CovVe1Ve2 is the sampling covariance between Ve1 and Ve2, and CovVe1Ce is the sampling covariance between Ve1 and Ce

**Table 3:** Bivariate twin model-fitting results (with standard errors) for general cognitive ability (‘g’) versus language, mathematics, and reading, as well as comparison data for: g and height, g and weight, and height and weight

Variables	A				C				E				n/pairs
	V(G)_tr1	V(G)_tr2	C(G)_tr12	r_G_	V(c)_tr1	V(c)_tr2	C(c)_tr12	r_C_	V(e)_tr1	V(e)_tr2	C(e)_tr12	r_E_	
‘g’ vs language	0.47(0.05)	0.41(0.05)	0.36(0.04)	0.80(0.06)	0.21(0.05)	0.22(0.04)	0.19(0.03)	0.90(0.10)	0.33(0.02)	0.37(0.02)	0.09(0.01)	0.27(0.03)	2205
‘g’ vs maths	0.46(0.05)	0.48(0.04)	0.36(0.03)	0.73(0.03)	0.21(0.04)	0.20(0.04)	0.19(0.03)	1.0(0.10)	0.33(0.02)	0.32(0.02)	0.07(0.01)	0.23(0.03)	2095
‘g’ vs reading	0.46(0.05)	0.59(0.04)	0.34(0.03)	0.66(0.05)	0.21(0.04)	0.17(0.04)	0.15(0.03)	0.85(0.12)	0.33(0.02)	0.24(0.01)	0.06(0.01)	0.20(0.04)	2104
‘g’ vs height	0.48(0.05)	0.80(0.04)	−0.02(0.03)	−0.03(0.06)	0.19(0.04)	0.10(0.04)	0.08(0.03)	0.54(0.23)	0.33(0.02)	0.10(0.01)	0.01(0.01)	0.07(0.04)	1716
‘g’ vs weight	0.48(0.05)	0.83(0.03)	−0.04(0.04)	−0.06(0.06)	0.19(0.04)	0.05(0.03)	0.03(0.03)	0.32(0.51)	0.33(0.02)	0.12(0.01)	0.03(0.01)	0.13(0.04)	1716
Height vs weight	0.81(0.04)	0.85(0.04)	0.54(0.03)	0.65(0.02)	0.09(0.04)	0.04(0.02)	0.06(0.03)	1.0(0.00)	0.10(0.01)	0.11(0.01)	0.06(0.01)	0.41(0.03)	2162

OpenMx twin model-fitting incorporates full-information maximum likelihood that uses the full sample of more than 2,000 pairs of twins with data on trait 1 or trait 2. However, the variance estimates for each trait are based on individuals with data for that trait. The covariance estimates are based on twin pairs with data for both traits, which is the conservative sample size shown in the last column

*V(G)* proportion of the variance explained by genetic factors for trait 1 and trait 2 (tr1, tr2), *C(G)* proportion of the covariance between trait 1 and 2 explained by genetic factors; *V(c)* proportion of the variance explained by shared environment for trait 1 and trait 2 (tr1, tr2), *C(c)* proportion of the covariance between trait 1 and 2 explained by shared environment, *V(e)* proportion of the variance explained by non-shared environment for trait 1 and trait 2 (tr1, tr2), *C(e)* proportion of the covariance between trait 1 and 2 explained by non-shared environment, *rG* genetic correlation; *rC* correlation of shared environmental factors; *rE* correlation of non-shared environmental factors, *n* number of twin pairs with data for both trait 1 and trait 2; values in parentheses are standard errors

## Discussion

Using DNA evidence alone, these high genetic correlations estimated from GCTA support the Generalist Genes Hypothesis in showing strong pleiotropy between ‘g’ and learning abilities, especially because we show that these GCTA-estimated genetic correlations are as high as genetic correlations estimated from the twin design.

Although GCTA does not identify specific genes associated with these traits, it addresses a critical issue in genome-wide association studies: the extent to which common SNPs used on commercially available SNP arrays can account for the heritability of quantitative traits (Yang et al. 2011b). We have shown in univariate GCTA analyses that, if samples were sufficiently large, common SNPs could account for more than two-thirds of the heritability of cognitive abilities estimated in twin studies (Yang et al. 2011b; see also Table 2). Why are univariate GCTA heritability estimates less than the twin study estimates of heritability? As discussed elsewhere (e.g. Yang et al. 2010), the main problem is imperfect tagging. The common SNPs used on all available commercial arrays only capture what is in LD with them. Rare variants, which have lower minor allele frequency, will thus not be ‘tagged’ and their influence will be missed. In addition, GCTA estimates additive genetic influence only, so that non-additive effects (gene–gene and gene-environment interaction) are not captured either.

A more novel question, and central to the present paper, is why, as we have shown here, bivariate genetic correlations estimated by GCTA are as great as twin study estimates. The likely reason is that attenuation of the estimated additive genetic variance due to imperfect linkage disequilibrium between causal variants and genotyped SNPs applies to both the additive genetic variance of the two traits and to their additive genetic covariance by the same proportion. Thus, the GCTA estimate of the genetic correlation is unbiased because it is derived from the ratio between genetic covariance and the genetic variances of the two traits.

Are generalist genes all in the mind (cognition) or are they in the brain as well? That is, genetic correlations between cognitive and learning abilities might be epiphenomenal in the sense that multiple genetically independent brain mechanisms could affect each ability, creating genetic correlations among abilities. However, the genetic principles of pleiotropy (each gene affects many traits) and polygenicity (many genes affect each trait) lead us to predict that generalist genes have their effects further upstream, creating genetic correlations among brain structures and functions, a prediction that supports a network view of brain structure and function.
